# Effectiveness of sensory electrical stimulation augmented virtual reality training on sitting balance and quality of life in individuals with incomplete spinal cord injury: study protocol for a randomized controlled trial

**DOI:** 10.1186/s13063-026-09837-7

**Published:** 2026-06-15

**Authors:** Nikhil Chowdhary, Stuti Khanna, Garima Wadhwa, Shefali Walia

**Affiliations:** 1 Institute of Rehabilitation Sciences, Indian Spinal Injuries Centre, New Delhi, India; 2 Department of Physiotherapy, Gurugram University, Gurugram, Haryana India

**Keywords:** Spinal cord injury, Sitting balance, Quality of life, Virtual reality, Sensory electrical stimulation, Rehabilitation

## Abstract

**Background:**

Individuals with incomplete spinal cord injury (iSCI) often face significant impairments in sitting balance and mobility due to trunk instability. These challenges can severely impact functional abilities, participation in daily activities, and overall quality of life (QoL). Studies have demonstrated the potential of virtual reality (VR) training to improve sitting balance in individuals with iSCI. Furthermore, sensory electrical stimulation (SES) has shown positive effects on sitting balance in various neurological populations. However, research exploring the combined effectiveness of virtual reality and sensory electrical stimulation on sitting balance and QoL, specifically within the iSCI population, remains limited.

**Methodology:**

This study will be an assessor-blind, parallel, two-group, randomized controlled trial that aims to evaluate the effectiveness of SES-augmented VR-based balance training on sitting balance and QoL in individuals with iSCI. A total of 22 participants with iSCI, with a neurological level of injury between T6 and T12, will be recruited from the rehabilitation department at the Indian Spinal Injuries Centre, adhering to inclusion criteria. Participants will be randomly assigned to one of two groups using a 1:1 allocation ratio. The experimental group will engage in VR-based balance training along with SES application, while the control group will engage solely in VR-based balance training. Both groups will receive interventions consisting of 30-min sessions five times a week for a duration of 4 weeks.

**Outcome measures:**

The assessment of sitting balance will be done using the modified functional reach test (mFRT) and the star test using the Tecnobody ProKin 252 trunk sensor. Additionally, the International Spinal Cord Society Quality of Life Basic Data Set (ISCoS QoL BDS) version 1.0 will be used to measure QoL.

**Discussion:**

The effectiveness of the SES-augmented VR-based balance training will be evaluated based on the changes in the mFRT, star test (ProKin 252 trunk sensor), and ISCoS QoL BDS version 1.0 following 4 weeks of intervention. This trial aims to enhance understanding of how SES-augmented VR training can improve sitting balance and QoL in individuals with iSCI.

**Trial registration:**

The trial is registered with the Clinical Trials Registry—India on 18th April 2024 with registration number CTRI/2024/04/065897.

**Supplementary Information:**

The online version contains supplementary material available at 10.1186/s13063-026-09837-7.

## Background

Spinal cord injury (SCI) is a debilitating neurological condition characterized by sensory, motor, autonomic, or bowel dysfunction caused by damage to neuronal elements of the spinal cord. Its incidence ranges from 8 to 246 cases per million worldwide, whereas the prevalence ranges from 236 to 1298 per million [1], with approximately 20,000 new cases documented annually in India [2]. Countries such as India are at high risk, with an average age group ranging from 26.8 to 56.6 years with a male-to-female ratio of 4:1 [1]. It often leads to a profound disability that negatively impacts physiological, physical, or psychological well-being and restricts employment opportunities even after the individual has reintegrated into the community.

Following SCI, maintaining sitting balance is essential for performing activities of daily living. Approximately 70–80% of individuals with SCI experience impaired or no trunk control, which adversely affects their ability to maintain sitting balance and overall quality of life (QoL) [3]. Trunk instability is a significant concern for individuals with SCI [4], as it impacts their ability to perform everyday tasks such as bed movements, unsupported sitting, and self-care activities [5, 6]. Additionally, it can lead to secondary complications such as pressure sores [7], spinal deformities [8], and pulmonary dysfunction [5]. Furthermore, individuals with SCI are also at a higher risk of falls even during stationary sitting, leading to fall-related pain and bone fractures [9]. Consequently, QoL in individuals with SCI is closely linked to their dynamic sitting balance and ability to sit unsupported [10], a relationship that becomes increasingly important over time post-injury [8].

Given these challenges, recovery of trunk control is essential for those experiencing difficulties with sitting balance [11]. However, research investigating sitting balance in individuals with incomplete spinal cord injury (iSCI) remains limited [10]. Conventional methods for maintaining sitting stability often involve belts, straps, and customized seating adaptations designed to stabilize the trunk, prevent falls, and facilitate upper limb function [12]. Unfortunately, these techniques can restrict dynamic mobility and hinder the ability to reach for objects [13].

In individuals with iSCI at thoracic levels T6–T12, injury to the descending motor tracts disrupts supraspinal control of key trunk musculature, impairing the voluntary activation of erector spinae and other postural muscles essential for maintaining sitting posture. At the same time, damage to ascending sensory pathways also reduces proprioceptive and cutaneous afferent input, impairing both anticipatory postural adjustments preceding voluntary movement and reactive responses necessary to counter external perturbations. The combined impairment of descending motor drive and ascending sensory feedback at these thoracic levels results in a deficit in neuromuscular control [14, 15].

The recovery of sitting balance is a primary objective in rehabilitation programs for individuals with iSCI. While research on effective balance training protocols for individuals with iSCI is scarce, recent advancements and the existence of many strategies like electrical stimulation [16], non-invasive spinal stimulation [9], community exercise programs [17], functional electrical stimulation [18], and virtual reality (VR) [19, 20] demonstrate improvement in mobility along with postural control. Methods utilizing VR have demonstrated positive results for training balance across various neurological populations. This technology provides a user-computer interface that facilitates real-time simulation of activities or environments, allowing user interaction through various sensory modalities and enhancing engagement and motivation during rehabilitation. Previous studies utilizing game-based exercises within VR have shown improvements in dynamic sitting balance among individuals with SCI [21–23].

Sensory electrical stimulation (SES) involves the use of low-intensity currents (below, at, or only just above the sensory threshold), which does not induce any visible muscle contraction and provides only sensory information. The beneficial effects of SES are thought to occur at the peripheral (sensory receptor sensitivity), spinal (spinal motoneural excitability), and supraspinal (cortex reorganization or adaptation) levels. SES appears to be particularly useful to reinforce or restore the postural function in the immediate/concurrent, acute, or chronic applications in pathological populations. Studies have already shown that SES is effective at improving postural balance in various neurological conditions, such as stroke and cerebral palsy [24, 25].

The integration of VR and SES may address the deficit in neuromuscular control. VR promotes neuroplasticity through enriched, multisensory, goal-directed motor tasks that engage residual pathways, reinforce motor relearning, and provide augmented visual and proprioceptive feedback within task-specific environments [19, 20]. Concurrently, SES enhances peripheral afferent input and modulates spinal excitability, thereby facilitating sensorimotor reorganization [24, 25]. When delivered simultaneously, these modalities may act synergistically, with SES amplifying afferent sensory input and VR providing a task-specific motor context, thereby facilitating neuroplastic adaptations.

Therefore, this study aims to investigate the effectiveness of SES-augmented VR-based balance training on sitting balance and QoL in individuals with iSCI. It is hypothesized that significant improvement in sitting balance and QoL will be noticeable after 4 weeks of SES-augmented VR-based balance training when compared with participants receiving VR-based balance training alone.

## Materials and methods

A clinical trial is designed to evaluate the effectiveness of the SES-augmented VR-based balance training on sitting balance and QoL in individuals with iSCI.

### Patient and study design

An assessor-blind, parallel, two-group, randomized controlled trial with equal subject allocation (1:1) will be undertaken. A sample of 22 participants with iSCI will be recruited from the inpatient and outpatient rehabilitation departments of the Indian Spinal Injuries Centre, New Delhi, India. All participants will be provided with information sheets, and written consent will be obtained by the principal investigator before recruitment. The demographic details will be obtained, and the participants will be selected based on the eligibility criteria after neurological examination (Table 1).

**Table 1:** Eligibility criteria

	Criteria
Inclusion criteria	Diagnosis of spinal cord injury > 6 months T6–T12 level of injury ASIA B, C, D Age between 18 and 60 years Ability to sit unsupported for 10 s and a minimum of 90° shoulder flexion
Exclusion criteria	Uncorrected hearing or visual impairment that interferes with the ability to perceive visual stimuli, follow verbal instructions, or safely participate in the virtual reality-based intervention Deformity of upper limb, any other psychiatric, neurological, or musculoskeletal impairment History of severe recent urinary tract infection or autonomic dysreflexia Refusal or inability to provide informed consent and inability to follow commands Additional exclusion criteria (sensory electrical stimulation group): 1. Pressure sores ≥ grade 2 at the site of stimulation according to National Pressure Ulcer Advisory Panel Classification 2. Cardiac pacemaker

The participants will be randomly assigned to any of the two groups—the virtual reality-based balance training along with sensory electrical stimulation (VR-SES) group and the virtual reality-based balance training (VR) group. Both groups will receive intervention sessions five times per week for 4 weeks.

**Fig. 1:**
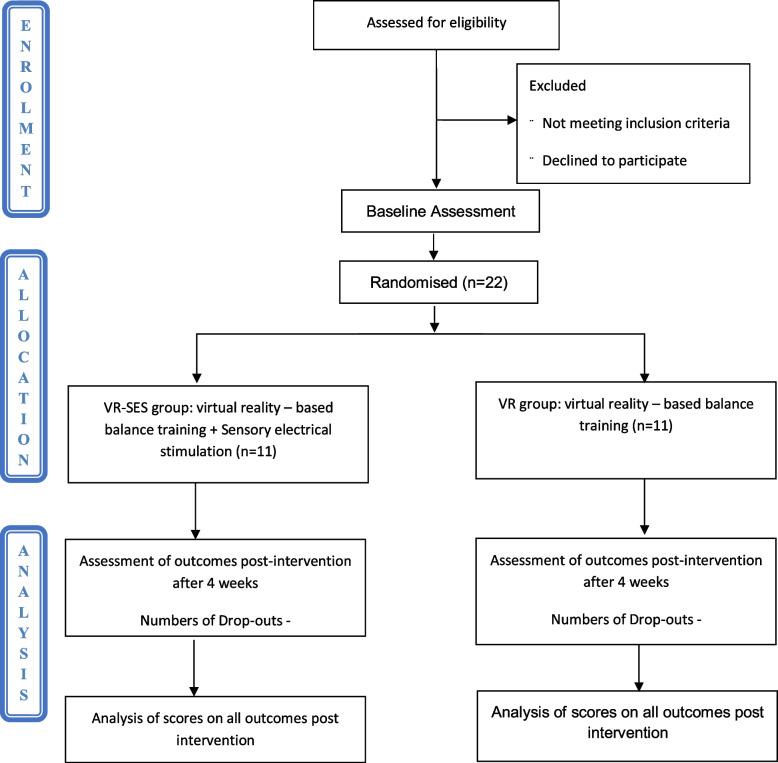
CONSORT (Consolidated Standards of Reporting Trials) flowchart. VR-SES group, virtual reality-based balance training along with sensory electrical stimulation; VR group, virtual reality-based balance training

The Departmental Research Review Committee (DRRC) and Research Review Committee (RRC) of the research department of the Indian Spinal Injuries Centre hold the legal liability and keep the check and record of all potential recruits and monitor the data collection throughout the intervention. The members of DRRC, along with the consultants and physiotherapists in the hospital, provide day-to-day support to all aspects of the local organization of the trial. The day-to-day conduct of the trial, including participant recruitment, monitoring, intervention implementation, and protocol adherence, will be overseen by the principal investigator under the supervision of the guides. The Trial Management Group (TMG) will conduct meetings monthly to ensure oversight of trial progress and operational issues. The DRRC will conduct audits of the trial every 6 months, while the Institutional Ethics Committee (IEC) will undertake an annual review to ensure compliance with the approved protocol and ethical standards. A Data Monitoring Committee (DMC) will not be constituted, as this study involves a low-risk intervention. This research protocol is consistent with the current Consolidated Standards of Reporting Trials (CONSORT) guidelines (Fig. 1) and follows the Standard Protocol Items: Recommendations for Interventional Trials (SPIRIT schedule) (Table 2) [26] and is developed based on the SPIRIT checklist (Additional document 1). A visual description of the study regarding enrollment, assessments, and interventions is shown in Table 2.

**Table 2:**
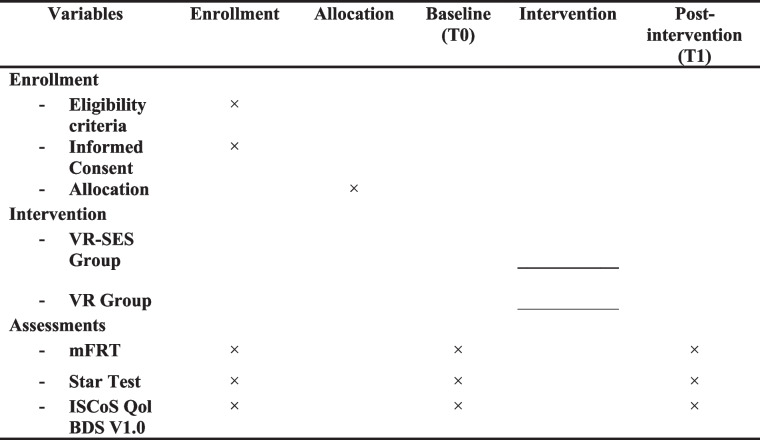
Schedule of enrollment, interventions, and assessments according to the Standard Protocol Items: Recommendations for Interventional Trials guideline

*Abbreviations*: *VR-SES group* Virtual reality-based balance training along with Sensory electrical stimulation, *VR group* Virtual reality-based balance training, *mFRT* Modified functional reach test, *ISCoS Qol BDS V1.0* International Spinal Cord Society Quality of Life Basic Data Set Version 1.0.

Baseline assessments (T_0_) will be done before group allocation. Post-intervention assessments of both groups (T_1_) will be done after 4 weeks of intervention to detect potential long-term effects.

### Ethical considerations

The enrolled participants will be informed orally and in writing about the purpose of this trial, its potential risks, the benefits of participation, and their right to withdraw from the trial at any point during the study. A written informed consent signed by the participants will be taken from those who are willing to participate in the study.

The data of the participants will be collected, documented, and managed confidentially using the paper-based entry data sets. Only all the authors related to the trial will have access to the final trial dataset. The masked datasets analyzed during the current study and statistical code will be available from the corresponding author upon reasonable request. The study protocol has been approved by the IEC of the Indian Spinal Injuries Centre and is registered with the Clinical Trials Registry—India (CTRI).

Any protocol deviations will be fully documented using a breach report form. In case of any important modification or amendment, the principal investigator will discuss the changes with the research team and the amendment will be formally documented and the revised protocol will be submitted to the RRC and IEC for approval prior to implementation. All amendments will also be updated in the CTRI.

Patients and the public will not be involved in the design, conduct, or reporting plans of this study.

### Randomization

Eligible individuals will be assigned randomly to either the experimental or control group. Randomization will be performed using a computer-generated randomization sequence generated by the Random Allocation Software, using a 1:1 allocation ratio.

To ensure concealment, the allocation sequence will be marked sequentially and sealed in opaque envelopes. An individual not associated with the study will sequentially open the numbered envelopes to reveal the participant’s group allocation.

### Blinding

The trial will be an assessor-blinded study where the outcome assessor, who will be a physiotherapist, will be blinded to group allocation. The principal investigator and participants will be informed of the group allocation given the nature of the interventions. Furthermore, the statistician who will perform data analysis would be unaware of the existence of treatment groups.

### Intervention

VR-based balance training will be provided by the Nintendo Wii Sports Resort (Nintendo Co. Ltd., Kyoto, Japan). It uses a central game console connected to a 127-cm (50-inch), wall-mounted television screen. Navigation is via the handheld remote control in the gaming environment. The choice of games was based on the similarity between the training and real-life conditions and based on previous research using the Nintendo Wii in training sitting balance in various neurological populations. During the first session, the therapist will demonstrate the game controls and instructions to ensure that the participants understand how to control the system. Furthermore, the therapist will offer verbal and manual guidance to all participants to help them learn the best strategies to reach the highest scores in each game. An equal number of rounds per exercise will be presented to the participants during each training session. Rest would be allowed and training would be resumed afterward if the participant declared fatigue (Table 3).

**Table 3:** Description of games using Nintendo Wii Sports Resort program

Game	General description
Table tennis	Sitting with the Nintendo Wii remote in hand, subjects must hit the ball in the plane of the table and prevent it from falling off the table in a game of 6 points
Swordplay	Sitting with the Nintendo Wii remote in hand, subjects must slice out different objects shown on the screen following directional cues marked on them
Bowling	Sitting with the Nintendo Wii remote in hand, subjects mimic the motion of bowling by moving their arms sideways and performing a free, fluid throwing motion aimed at knocking down all the pins in a simulated bowling lane
Golf	Sitting with the Nintendo Wii remote in hand, subjects mimic; there is a map and speed and angle chart on the screen, seeing which they must target the ball in the hole by hitting it with the stick by managing the force, and the fewer the number of hits, the higher the score
Frisbee	Sitting with the Nintendo Wii remote in hand, subjects visualize it as a frisbee and try to throw it toward a marked arrow displayed on the screen, with the goal of landing as close to the arrow as possible to achieve the highest score
Canoeing	Sitting with the Nintendo Wii remote in hand, subjects mimic the motion of paddling by changing the direction of the remote to navigate straight ahead with fluid hand movements. The objective was to reach the final target within 1 min while maintaining a steady rhythm and control

Participants in the VR-SES group will receive SES while performing virtual reality-based balance training. SES will be delivered using a transcutaneous electrical nerve stimulation (TENS) machine (HMS DigitensII) that uses surface self-adhesive stimulation electrodes placed on the subject’s skin corresponding to the muscles targeted. The key muscles that will be targeted are the erector spinae.

**Fig. 2:**
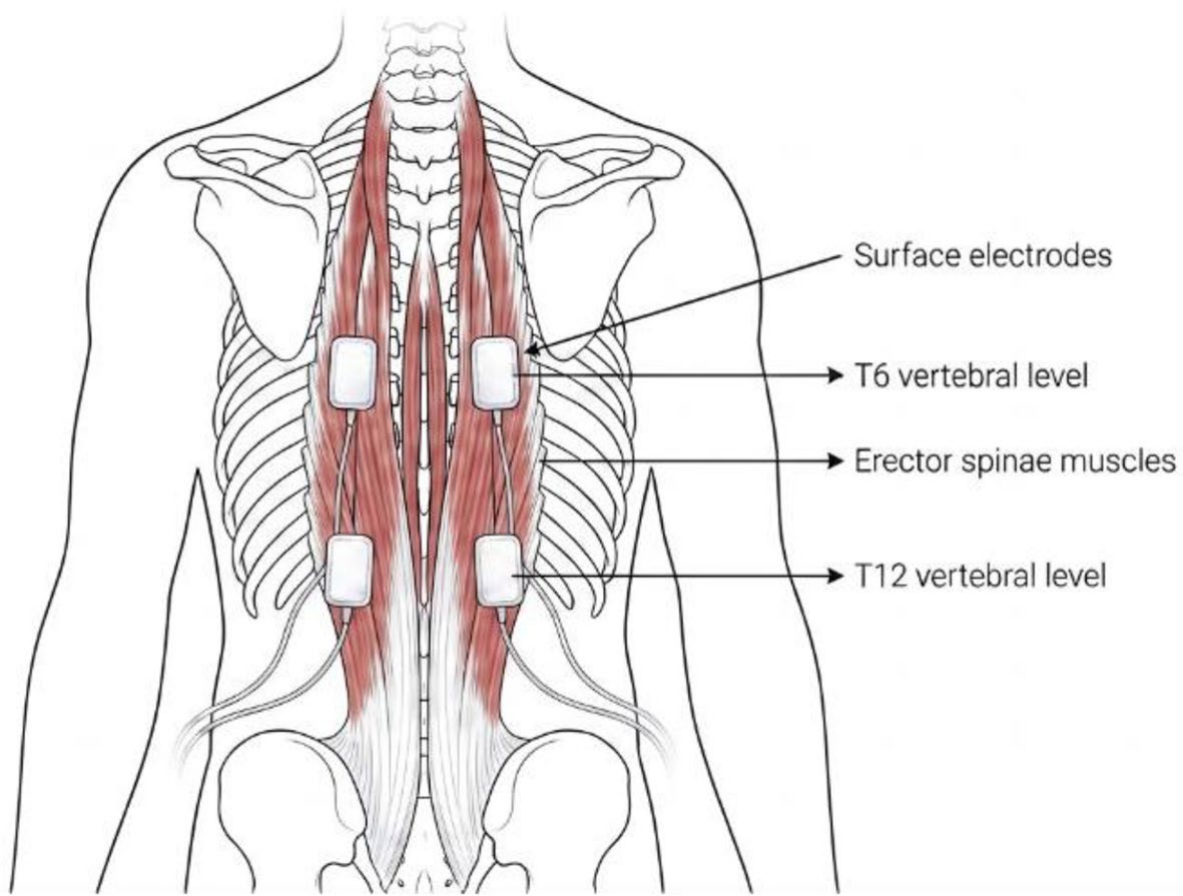
Schematic representation of electrode placement for sensory electrical stimulation (SES) over the erector spinae muscles

Electrodes will be placed bilaterally on the muscle belly of the erector spinae in the thoracolumbar region, along the orientation of the muscle fibers at the T6 and T12 vertebral levels (Fig. 2). SES will be applied continuously throughout the entire 30-min VR training session.

The pulse width will be 200 μs, and the pulse frequency will be 100 Hz. The current levels will be separately selected such that the participant feels the stimulation at the level (suprasensory threshold) without having the stimulated muscles contract due to the stimulation [27]. The suprasensory threshold will be determined by gradually increasing the stimulation intensity until the participant first perceives the stimulus, and then further increasing it to a clearly perceptible, comfortable level without any visible or palpable muscle contraction, as confirmed by the therapist’s observation. This process will be followed for all participants, and all sessions will be administered by a single trained therapist to ensure consistency of the sessions. Adverse events will be monitored and reported, if any.

### Progression and feedback

The initial difficulty will be adjusted for each participant based on their performance during the familiarization session. Progression of task difficulty will be based on predefined game-specific performance indicators generated by the Nintendo Wii, including in-game scores, task completion, and stage progression. Participants will receive concomitant visual and auditory feedback regarding task performance when movements are accurately performed, as learning is also affected by performance-related feedback. Participants will also receive negative feedback about their performance when they use compensatory strategies while playing games on the Nintendo Wii verbally by the supervising therapist.

### Termination criteria

The intervention will be terminated if any kind of unusual uneasiness is reported by the participants. Participants will be instructed to report any adverse events experienced during or after the intervention throughout the study period to the principal investigator or research team. The intervention session will be discontinued if the participants report discomfort, pain, or excessive fatigue during the session.

### Adherence

The adherence of the participants recruited in the study will be monitored by documenting the details of sessions attended. Participants in all groups will receive VR-based balance training, which may positively influence adherence and reduce attrition rates, as all groups are likely to feel engaged in an active intervention.

The participants will be permitted to increase the training duration by 1 week, keeping in mind the “intention-to-treat” analysis principle if the participants are unable to complete the total number of sessions within the stipulated 4 weeks. Also, the authors will fully report the type, extent, and pattern of missing data throughout the study (Table 4).

**Table 4:**
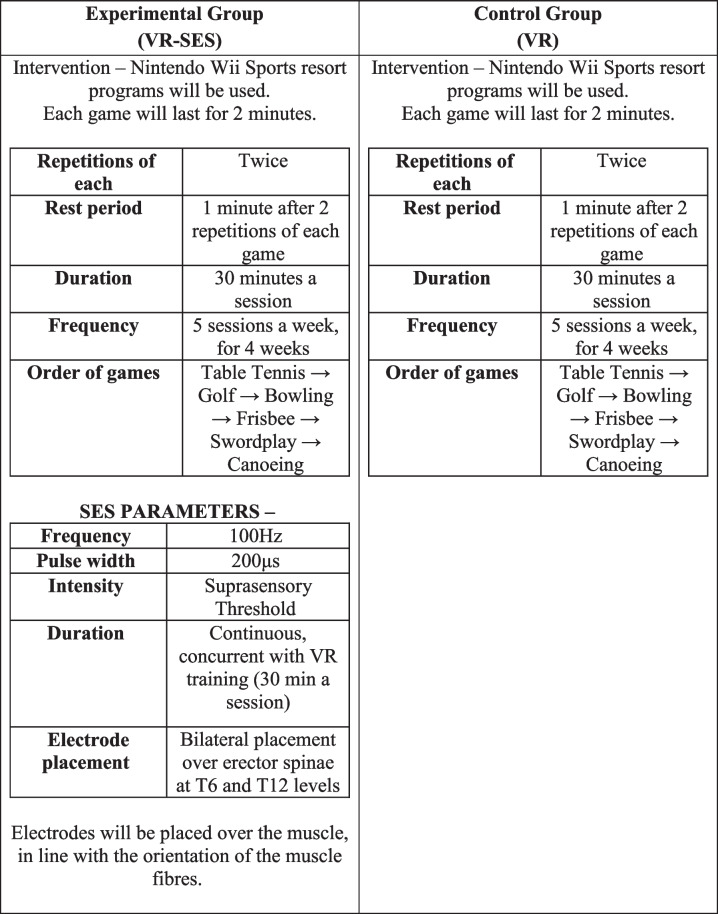
Protocol for experimental (VR-SES) and control (VR) group

### Outcome variables

Modified functional reach test (mFRT): The participant sits in a standard wheelchair, with hips, knees, and ankles positioned at approximately 90° and feet supported. Initial reach is measured against a yardstick mounted on the wall, and then the maximum forward reach is measured using the ulnar styloid process as a landmark. The difference is the measure of functional reach in a sitting position. Each participant performs three trials, and the average of these measurements is used for analysis. It has been reported to have good to excellent reliability, with intraclass correlation coefficient (ICC) values ranging from 0.78 to 0.99 [28].

Star test: The ProKin trunk sensor platform (ProKin PK252; TecnoBody Inc., Dalmine, Italy) will be used. The participant sits on the ProKin 252 multiaxial platform with their back straight, hip and knee flexed at 90°. During the star test, a target appears in different directions on the screen. The participant has to reach the target on a given path by shifting his weight toward that direction. The shifting of weight is done by moving the multiaxial platform in different directions. After a series of such movements, the Tecnobody software provides a final report in terms of total area covered during the test. It has been found that the ProKin device is reliable and valid for assessing standing balance in individuals with iSCI. Test-retest reliability was found to be moderate to excellent (ICC = 0.64–0.93) [29].

International SCI Quality of Life Data Set—version 1.0: It was developed as a brief QoL measure for use in clinical practice and research. It consists of three items for an individual to rate their satisfaction with life, physical health, and psychological health. Each domain is ranked on a 0–10 scale, where 0 indicates complete dissatisfaction and 10 indicates complete satisfaction. The tool has been reported to be both valid and reliable. The three items exhibited strong inter-correlations, ranging from 0.48 to 0.71. Furthermore, the scale demonstrated good internal consistency with a Cronbach’s alpha of 0.81, and item-rest correlations fell between 0.57 and 0.74 [30].

## Statistics

### Sample size

A priori sample size estimation was done using the G*Power 3 software (3.1.9.6, Heinrich-Heine-Universität Düsseldorf, Düsseldorf, Germany) by considering the mFRT as the primary outcome measure. The total sample size was calculated at 20 by using an *α* level of 0.05 and a power of 80%. The potential loss to attrition was estimated at 10%. Hence, the total sample size was 22.

### Statistical analysis

To check for selection bias, we will apply Pearson’s chi-square test for categorical variables and the Student *t*-test for numerical variables. This will be performed to know if the randomization process generated between each group of participants has homogeneous clinical and demographic characteristics before the intervention.

Continuous variables will be summarized as mean ± standard deviation for normal distribution and median ± interquartile range for non-normal distribution. The Shapiro-Wilk test will be used to determine the normality of data. In the case of normal distribution of data, we will analyze the intergroup comparison by applying a two-sample *t*-test. In case of skewed data, we will use the Mann-Whitney *U* test for the intergroup comparison. The intragroup comparison will be analyzed by repeated-measure analysis of variance (ANOVA) or Friedman’s test (if data is not normally distributed). The final statistical analysis will be performed using IBM SPSS Ver. 29.0 (IBM Corp., Armonk, NY, USA). This will be done based on the modified intention-to-treat principle, whereby each participant must complete > 75% (15 of 20 sessions) of planned exercise sessions. The principle of the “last observation carried forward” will be applied in the case of missing data for dropped-out individuals. The level of statistical significance is assumed at a *p *value < 0.05. No interim analysis will be conducted due to the short duration of the study. Formal stopping rules based on statistical criteria have not been defined. However, the trial may be terminated early upon recommendation of the IEC in case of safety concerns or unforeseen circumstances.

## Discussion

The recovery of sitting balance is one of the primary and essential aims of rehabilitative programs in individuals with iSCI. Retraining sitting balance is challenging for healthcare professionals dealing with iSCI. The trial will provide information about the effectiveness of SES-augmented VR-based balance training in improving sitting balance and QoL in individuals with iSCI. The training is expected to enhance sitting balance and QoL in individuals with iSCI. To the best of our knowledge, no study until now has examined the effectiveness of SES-augmented VR-based training on sitting balance and QoL in the iSCI population.

This trial is designed to meet the methodological demand for adequate randomization, allocation concealment, and blinding of outcome assessors and statisticians. The trial will be reported according to the CONSORT guidelines. This study has several limitations. The recruitment of the participants will be done through a sample from one center; it may limit the generalizability of the findings. The sample size, calculated based on the primary outcome measure, may limit the power to detect differences in secondary outcomes. Therefore, secondary outcome findings should be interpreted as exploratory and hypothesis-generating. The lack of long-term follow-up may limit the ability to assess the durability of the intervention effects. In future, multi-center trials incorporating stratified block randomization are recommended to strengthen comparability.

## Trial status

The second version of the original protocol after amendments was finalized on March 2, 2024. The first participant in the trial was recruited in August 2024, and the trial was completed in July 2025 as planned.

## Supplementary Information

Supplementary Material 1.

## Data Availability

Any data required to support the protocol can be provided on request by keeping the participant’s details confidential.

## Abbreviations

SCI: Spinal cord injury
iSCI: Incomplete spinal cord injury
QoL: Quality of life
VR: Virtual reality
SES: Sensory electrical stimulation
mFRT: Modified functional reach test
ISCoS QoL BDS: International Spinal Cord Society Quality of Life Basic Data Set
DRRC: Departmental Research Review Committee
RRC: Research Review Committee
IEC: Institutional Ethics Committee
DMC: Data Monitoring Committee
TENS: Transcutaneous electrical nerve stimulation
ICC: Intraclass correlation coefficient
ANOVA: Analysis of variance
